# Splenectomy Impact and Outcome Among Patients With Sickle Cell Disease: A Cross‐Sectional Study

**DOI:** 10.1002/hsr2.72778

**Published:** 2026-07-07

**Authors:** Mortadah Alsalman, Zainab Bu‐Khamsin, Fatimah AlSahaf, Fatima Al Amer, Fatema Alhelal, Ali Essa, Mohammed Alessa, Somaia Shehab‐Eldeen, Abdallah Essa

**Affiliations:** ^1^ Department of Medicine, College of Medicine King Faisal University Al Ahsa Saudi Arabia; ^2^ College of Medicine King Faisal University Al Ahsa Saudi Arabia; ^3^ Department of Family and Community Medicine Al Ahsa Health Cluster Al Ahsa Saudi Arabia; ^4^ Department of Intensive Care Mouwasat Hospital Saudi Arabia; ^5^ Faculty of Medicine Menoufia University Shebin Elkom Egypt; ^6^ Department of Surgery, College of Medicine King Faisal University Al Ahsa Saudi Arabia; ^7^ Tropical Medicine Department, Faculty of Medicine Menoufia University Shebin El‐kom Egypt

**Keywords:** clinical outcomes, pain crisis, Saudi Arabia, sickle cell disease, splenectomy

## Abstract

**Background:**

It is common to perform a splenectomy for patients with sickle cell disease (SCD); however, our understanding of its impact on long‐term clinical outcomes remains incomplete.

**Objectives:**

To evaluate the clinical outcomes of splenectomy in SCD patients and to assess perioperative physician practices.

**Methods:**

This is a retrospective cross‐sectional study that involved 245 adult Saudi patients with SCD, categorized into two groups: 40 patients who underwent splenectomy and 205 who did not. Data were gathered by structured interviews and electronic health records, including sociodemographic information, features of SCD, surgical procedures, and post‐splenectomy outcomes.

**Results:**

The splenectomy group exhibited an increased frequency of acute chest syndrome (40% vs. 8.3%) and annual hospital admissions (8.7 vs. 3.35) due to pain crises before splenectomy. Following splenectomy, they demonstrated a notable decrease in hospital admissions and the need for blood transfusions (92.5% vs. 97.1%, 82.5% vs. 84.9%, respectively). Nevertheless, they exhibited a greater prevalence of stroke, venous thrombosis, and acute chest syndrome compared to the non‐splenectomy group (15% vs. 2.4%, 12.5% vs. 2.9%, and 45% vs. 8.3%). Patients aged ≤ 33 years (*p* = 0.03) and those with comorbidities (*p* = 0.02) demonstrated a significant response to splenectomy.

**Conclusion:**

Splenectomy in SCD patients may result in reduced hospital admissions and blood transfusion needs. Yet, it may elevate the risk of thromboembolic events and acute chest syndrome. Optimizing outcomes require careful patient selection and perioperative management.

## 1 Introduction

Sickle cell disease (SCD) is one of the most common autosomal recessive genetic disorders worldwide. The disease's global birth incidence has shown a 13.7% increase between 2000 and 2021 [1, 2]. Saudi Arabia has a high prevalence rate of SCD affecting 20,000 people per 1,000,000. Two distinct haplotypes exist in Saudi Arabia; the Arab‐Indian (AI) and the Benin haplotypes predominate in the Eastern and the Southwestern Provinces, respectively [3].

Sickle cell disease consists of a group of hemoglobinopathies in which individuals inherit hemoglobin variants derived from single point mutations resulting in sickling of red blood cells, hemolysis, inflammation, and tissue ischemia. The complex interplay between these various pathophysiologies and molecular genotypes leads to multi‐systemic disease with a broad range of acute and chronic clinical complications [4, 5]. The majority of complications are manageable, though they generate a significant economic burden on society, and the healthcare system and affect patients' quality of life, as evidenced by frequent emergency visits, readmission rates, and prolonged hospitalization [4, 6].

SCD is potentially curable after allogeneic hematopoietic stem cell Transplantation (HSCT) or autologous HSCT with gene therapy. Both treatment options seek to fully eradicate disease complications and improve life expectancy; however, the cost of the gene therapy procedure, a lack of suitable donors and post‐transplant complications may limit the broad use of these therapies [7, 8]. Therefore, the current treatment paradigm for patients with SCD relies on supportive care to manage acute complications, reduce disease severity, and possibly prolong survival as a bridge to curative treatment options [8, 9]. A variety of surgical and non‐surgical therapeutic options are available, including disease‐modifying therapies like hydroxyurea and simple or exchange blood transfusions, immunization, and splenectomy [4, 10]. Spleen complications among patients with SCD are serious, such as acute splenic sequestration (ASS) or hypersplenism and may serve as a marker of severity. Furthermore, the prevention of these complications is uncertain despite the utilization of preventive modalities, including disease‐modifying therapy and blood transfusion. On the other hand, the benefit‐to‐risk ratio of splenectomy can be challenging to define [10, 11]. Therefore, this study aims to study the splenectomy outcome among patients with SCD in comparison to non‐splenectomized individuals and evaluate peri‐procedural physician practice.

## 2 Methodology

### 2.1 Study Population

A retrospective cross‐sectional study on SCD patients at the Blood Disease Centre and King Faisal University in Al‐Ahsa, Saudi Arabia, was conducted from October 2022 to October 2024. The center specializes in hereditary blood diseases and is closely affiliated with the main hospital. It primarily serves patients with various hereditary blood disorders, including hemoglobinopathies and bleeding disorders. Patients are routinely monitored, and a range of services is offered, including scheduled blood transfusions. The inclusion criteria were Adult Saudi patients with SCD who are aged 18 years or more. The criteria for exclusion encompass individuals who have undergone splenectomy within the previous year. It is important to note that no patients were excluded from the study. Patients were recruited from the hospital's electronic database, where they were subsequently interviewed. We make every effort to select individuals who consistently attend follow‐up appointments and consent to participate, thereby ensuring the acquisition of complete laboratory data. The ethical committees at King Faisal University and King Fahad Hospital have granted approval for the study, with the respective ethical approval numbers KFU‐REC‐2022‐APR‐EA000582 and H‐05‐HS‐065. In addition, the participants were informed about the purpose of the research and the confidentiality of the data before giving their consent.

### 2.2 Data Collection

The data was collected by trained research members through direct interviews with patients using a structured questionnaire with four sections. The first section covered sociodemographic data such as age, gender, marital status, education level, and history of chronic disease. The second section incorporated SCD baseline characteristics, including the annual frequency of crises, the annual frequency of ER visits, previous history of acute chest syndrome, previous history of ICU admissions, previous history and frequency of blood transfusions, history of surgical intervention, and history of using hydroxyurea (duration and dose). The third section covered splenectomy‐related data such as vaccinations, type, and indications of the procedure. The fourth section was related to post‐splenectomy data, including SCD characteristics, including SCD crises, hospital admission, ICU admission, ER visits, and blood transfusion). The hospital's electronic database was used to collect patient's laboratory data, including complete blood count, hemoglobin electrophoresis, lactate dehydrogenase (LDH), and kidney function tests.

The Case Report Form (CRF)/structured questionnaire used for data collection is included as Supporting Information S1.

### 2.3 Statistical Analysis

Descriptive data were presented as mean ± standard deviation, median (range), or percentages. The comparison between patients who had splenectomy and those who did not was conducted using the *χ* ^2^ or Fisher's exact test for categorical variables and the Mann–Whitney U test for continuous data. The comparisons of the clinical data pre‐ and post‐splenectomy were done using the Wilcoxon matched‐pair single‐rank test for medians and McNemar's test for percentages. Linear and logistic regression analyses were used to identify predictors of favorable responses after splenectomy, using a 95% confidence interval (*p* < 0.05) to denote statistical significance. For age analysis, we divided it into two categories: age < 33 years and age > 33 years, since the median age for the splenectomy group was 33.5. All of the statistical analyses used two‐sided hypothesis testing with a significance threshold of *p* ≤ 0.05.

## 3 Results

**Figure 1:**
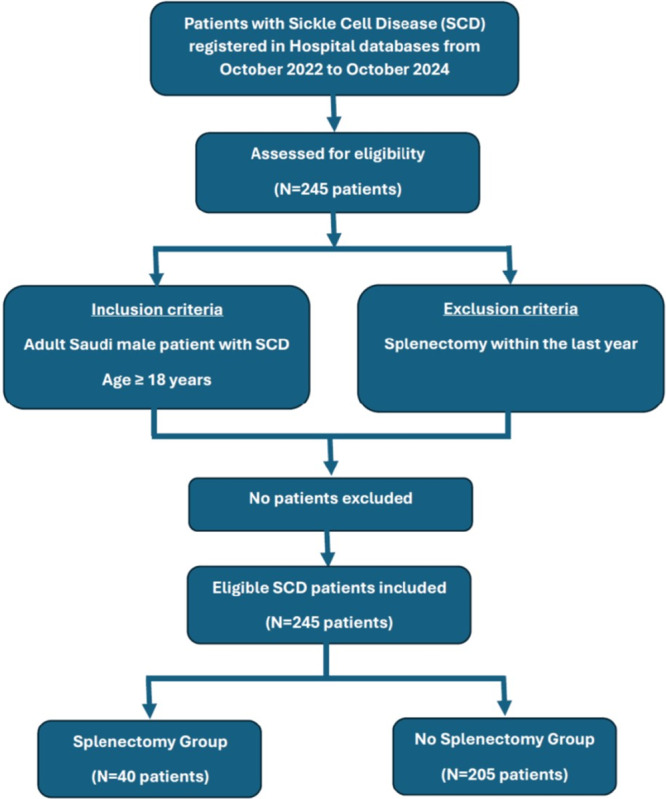
Data flow diagram.

The present study examined a cohort of 245 patients diagnosed with sickle cell disease. Of the 245 patients total, 40 were identified as having undergone splenectomy. Participants were categorized into two distinct groups: the splenectomy group, comprising 40 patients who had the procedure, and the no‐splenectomy group, which included 205 patients who did not undergo splenectomy (Figure 1). The mean age at which splenectomy was performed was 22 years. The analysis revealed significant differences between the two groups in terms of chronic illnesses. However, there were no notable differences concerning demographic factors such as age, gender, smoking status, level of education, or the use of disease‐modifying agents (Table 1).

**Table 1:** Patients' demographics.

Variable	Splenectomy *N* = 40	No splenectomy *N* = 205	*p* value
Age (mean ± SD)	37.3 ± 13.99	33.76 ± 11.64	
Median (range)	33.5 (19–74)	32 (18–88)	0.166^a^
Gender			
Male	19 (47.5%)	106 (51.7%)	0.73^b^
Female	21 (52.5%)	99 (48.3%)	
Smoking			
Smoker	10 (25%)	46 (22.4%)	0.7^b^
Non‐smoker	30 (75%)	159 (77.6%)	
Level of education			
Uneducated	3 (7.5%)	3 (1.5%)	0.16^b^
Primary	3 (7.5%)	15 (7.3%)	
Intermediate	2 (5%)	21 (10.2%)	
High school	17 (42.5)	75 (36.6%)	
Bachelor	15 (37.5%)	91 (44.4%)	
Chronic illnesses			
None	28 (70%)	162 (79%)	0.006^b^^,^ *
G6PD	8 (20%)	21 (10.24%)	
DM	2 (5%)	5 (2.44%)	
Hypertension	1 (2.5%)	8 (3.9%)	
Heart disease	2 (5%)	4 (1.95%)	
Chronic kidney disease	1 (2.5%)	3 (1.46%)	
Airway diseases	1 (2.5%)	3 (1.46%)	
SLE	1 (2.5%)	1 (0.5%)	
Rheumatoid arthritis	0 (0%)	1 (0.5%)	
Ulcerative colitis	0 (0%)	2 (1%)	
Usage of disease‐modifying agents			
Yes	9 (22.5%)	63 (30.7%)	
No	31 (77.5%)	142 (69.3%)	0.198^b^
Regular vaccination before splenectomy			
Yes	26 (65%)		
No	14 (35%)		
Regular vaccination after splenectomy			
Yes	22 (55%)		
No	18 (45%)		

^a^ Mann–Whitney U test.

^b^ *χ* ^2^ test.

* Significant at *p* ≤ 0.05.

A comparison of baseline characteristics highlighted marked differences between the groups. The splenectomy group exhibited a higher prevalence of acute chest syndrome and experienced more frequent crises requiring hospitalization each year (40% vs. 8.3%). Conversely, there were no significant differences with regard to hospital admissions, incidences of stroke, venous thrombosis, or the need for blood transfusions (92.5% vs. 97%, 0% vs. 2.4%, 2.5% vs. 2.9%, and 82.5% vs. 84.9%) (Table 2). Additionally, laboratory findings indicated no significant differences between the groups regarding hemoglobin electrophoresis parameters (Table 3).

**Table 2:** Baseline clinical characteristics of the studied groups.

Variable	Splenectomy	No splenectomy	*p* value
Hospital admission			
Yes	37 (92.5%)	199 (97.1%)	0.167^a^
No	3 (7.5%)	6 (2.9%)	
Number of crises/years that require hospital admission			
Mean ± SD	8.7 ± 9.1	3.35 ± 3.6	< 0.001^b^^,^ *
Median (range)	6 (0–50)	2 (0–24)	
History of stroke			
Yes	0 (0%)	5 (2.4%)	
No	40 (100%)	200 (97.6%)	
History of venous thrombosis			
Yes	1 (2.5%)	6 (2.9%)	0.88^a^
No	39 (97.5%)	199 (97.1%)	
History of acute chest syndrome			
Yes	16 (40%)	17 (8.3%)	< 0.001^a^^,^ *
No	24 (60%)	188 (91.7%)	
Blood transfusions			
Yes	33 (82.5%)	174 (84.9%)	0.07^a^
No	7 (17.5%)	31 (15.1%)	
Number of blood transfusions			
(mean ± SD)	9.5 ± 9.97	6.82 ± 8.5	0.134^b^
median (range)	6 (0–36)	(3–50)	

^a^ *χ* ^2^ test.

^b^ Mann–Whitney U test.

* Significant at *p* ≤ 0.05.

**Table 3:** Hemoglobin electrophoresis.

Variable	Splenectomy *N* = 40			No splenectomy *N* = 205			*p* value
	Median	Inter quartile range		Median	Inter quartile range		
		Percentile 25	Percentile 75		Percentile 25	Percentile 75	
Hb A1	7.4	0	12.97	0	0	10.5	0.1^a^
Hb A2	2.6	2	3.5	2.7	2.2	3.4	0.6^a^
Hb S	76.85	63.22	81.3	75	66.9	81.85	0.8^a^
Hb F	12.1	9.2	18.37	14.7	9	20.75	0.5^a^

Abbreviation: Hb, hemoglobin.

^a^ Mann–Whitney U test.

### 3.1 Splenectomy Procedure

A comprehensive overview of the surgical procedures performed reveals that 40 patients (19.5%) underwent splenectomy and spleens were completely resected. Of these, 52.5% received laparoscopic surgery, and 47.5% undergoing open surgery. Furthermore, 77.5% of the procedures were conducted electively, while 22.5% were classified as emergency surgeries. The primary indication for splenectomy was identified as chronic left upper quadrant pain, accounting for 32.5% of cases, followed by asymptomatic splenomegaly at 22.5%, and multiple blood transfusions associated with chronic left upper quadrant pain at 15% (Table 4).

**Table 4:** Details of splenectomy procedure in splenectomy group.

Variable	No. (%)
Indications of splenectomy	
Chronic left upper quadrant pain	13 (32.5%)
Asymptomatic large spleen	9 (22.5%)
Multiple blood transfusions and chronic left upper quadrant pain	6 (15%)
Multiple blood transfusions and asymptomatic large spleen	4 (10%)
Splenic abscess	4 (10%)
Multiple blood transfusion	1 (2.5%)
Recurrent painful crisis	1 (2.5%)
Splenic mass	1 (2.5%)
Previous sequestration crisis	1 (2.5%)
Type of procedure	
Complete	40 (100%)
Partial	0 (0%)
Technique of procedure	
Laparoscopic	21 (52.5%)
Open surgery	19 (47.5%)
Timing of the procedure	
Elective	31 (77.5%)
Emergency	9 (22.5%)

### 3.2 Clinical Outcomes of Both Groups

**Figure 2:**
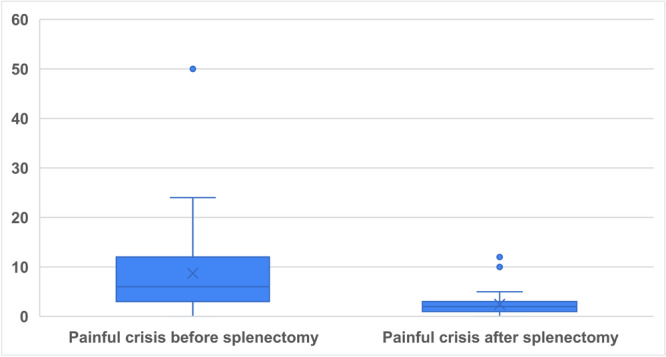
Painful crisis before and after splenectomy.

**Figure 3:**
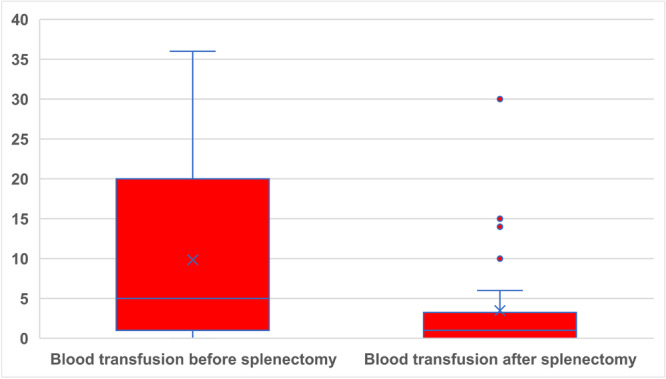
Blood transfusion before and after splenectomy.

Patients who underwent splenectomy experienced significantly fewer annual hospital admissions and blood transfusions after the procedure compared to those who did not have it. However, there was no significant difference between the two groups in the number of crises that required hospital admission each year. Contrarily, the rate of strokes, venous thrombosis, and acute chest syndromes was significantly higher among those who underwent splenectomy (Table 5). For patients who had splenectomy, there was a notable decrease in the number of crises per year that required hospitalization, as well as a reduction in the number of blood transfusions received post‐splenectomy when compared to the pre‐splenectomy period (Table 6, Figures 2, 3).

**Table 5:** Clinical outcomes of both groups.

Variable	Splenectomy	No splenectomy	*p* value
Hospital admission			
Yes	36 (90%)	199 (97.1%)	0.039^a^^,^ *
No	4 (10%)	6 (2.9%)	
Number of crises/years that require hospital admission			
Mean ± SD	2.45 ± 2.287	3.35 ± 3.6	0.323^b^
Median (range)	2 (0–12)	2 (0–24)	
History of stroke			
Yes	6 (15%)	5 (2.4%)	< 0.001^a^^,^ *
No	34 (85%)	200 (97.6%)	
History of venous thrombosis			
Yes	5 (12.5%)	6 (2.9%)	0.007^a^^,^ *
No	35 (87.5%)	199 (97.1%)	
History of acute chest syndrome			< 0.001^a^^,^ *
Yes	18 (45%)	17 (8.3%)	
No	22 (55%)	188 (91.7%)	
Blood transfusions			
Yes	28 (70%)	174 (84.9%)	0.024^a^^,^ *
No	12 (30%)	31 (15.1%)	
Number of blood transfusions			
Mean ± SD	3.45 ± 5.866	6.82 ± 8.5	0.003^b^^,^ *
Median (range)	1 (0–30)	(3–50)	

^a^ *χ* ^2^ test.

^b^ Mann–Whitney U test.

* significant at *p* ≤ 0.05.

**Table 6:** Clinical outcomes after splenectomy in the splenectomy group.

Variable	Before splenectomy	After splenectomy	*p* value
Hospital admission			
Yes	37 (92.5%)	36 (90%)	1^a^
No	3 (7.5%)	4 (10%)	
Number of crises/years that require hospital admission			
Mean ± SD	8.7 ± 9.1	2.45 ± 2.287	< 0.001^b^^,^ *
Median (range)	6 (0–50)	2 (0–12)	
History of stroke			
Yes	0 (0%)	6 (15%)	
No	40 (100%)	34 (85%)	
History of venous thrombosis			
Yes	1 (2.5%)	5 (12.5%)	0.219^a^
No	39 (97.5%)	35 (87.5%)	
History of acute chest syndrome			
Yes	16 (40%)	18 (45%)	0.815^a^
No	24 (60%)	22 (55%)	
Blood transfusions			
Yes	33 (82.5%)	28 (70%)	0.302^a^
No	7 (17.5%)	12 (30%)	
Number of blood transfusions			
Mean ± SD	9.5 ± 9.97	3.45 ± 5.866	< 0.001^b^, *
Median (range)	6 (0–36)	1 (0–30)	

^a^ McNemar's test.

^b^ Wilcoxon signed‐rank test.

* Significant at *p* ≤ 0.05.

### 3.3 Comparing Patients Who Underwent Splenectomy, Categorized According to Their Response

Our analysis reveals that patients aged 33 years or younger, along with those presenting comorbidities, demonstrated a markedly favorable clinical response following splenectomy (*p* = 0.03 and *p * = 0.02, respectively). Specifically, there was a reduction of 50% or more in the annual number of crises necessitating hospitalization. However, it is important to note that no significant differences were found between good and poor responders concerning gender, smoking status, utilization of disease‐modifying therapies, regular vaccination both prior to and following splenectomy, or results from hemoglobin electrophoresis (Table 7).

**Table 7:** Comparing patients who underwent splenectomy according to their response.

Variables	Good responders (*N* = 30)	Poor responders (*N* = 10)	*p* value
Categorical variables (*N*, %)			
Age ≤ 33	18 (60%)	2 (20%)	0.03^a^^,^ *
> 33	12 (40%)	8 (80%)	
Gender (No., %)			
Male	14 (46.7%)	5 (50%)	0.57^a^
Female	16 (35.3%)	5 (50%)	
Smoking			
Yes	9 (30%)	1 (10%)	0.204^a^
No	21 (70%)	9 (90%)	
Chronic illnesses			
Yes	24 (80%)	4 (40%)	0.02^a^^,^ *
No	6 (20%)	6 (60%)	
Usage of disease‐modifying agents			
Yes	7 (23.3%)	2 (20%)	0.83^a^
No	23 (76.7%)	8 (80%)	
Regular vaccination before splenectomy			
Yes	20 (66.7%)	6 (60%)	0.7^a^
No	10 (33.3%)	4 (40%)	
Regular vaccination after splenectomy			
Yes	17 (56.7%)	5 (50%)	0.71^a^
No	13 (43.3%)	5 (50%)	
Continuous variables (median, range)			
Hb A1	7 (0–46)	5.6 (0–50.6)	0.96^b^
Hb A2	2.5 (0–6)	3.6 (0–6.4)	0.14^b^
Hb S	78.1 (45.9–92)	68.9 (39.3–87)	0.131^b^
Hb F	12.1 (4.8–32)	13.05 (6.2–37)	0.135^b^

*Note:* The age categories (≤ 33 vs. > 33 years) were established based on the median age of the splenectomy group (33.5 years).

Abbreviation: Hb, hemoglobin.

^a^ *χ* ^2^ test.

^b^ Mann–Whitney U test.

* Significant at *p* ≤ 0.05.

### 3.4 Predictors Linked to Good Response to Splenectomy

We conducted both univariate and multivariate analyses to identify the factors associated with a favorable response to splenectomy. The univariate logistic regression analysis revealed two factors that were statistically significant: an age of 33 years or younger (odds ratio = 6; *p* = 0.04; 95% confidence interval = 1.08–33.3) and the presence of chronic illnesses (odds ratio = 6; *p* = 0.023; 95% confidence interval = 1.274–28.25). However, these factors did not retain their statistical significance as predictors when adjusted for other variables in the subsequent multivariate analysis (Table 8).

**Table 8:** Predictors of a good response to splenectomy.

Variable	Univariable analysis		Multivariable analysis	
	OR (95% CI)	*p* value	AOR (95% CI)	*p* value
Age (≤ 33)	6 (1.08–33.3)	0.04*	4.2 (0.69–25.34)	0.118
Sex (male)	0.875 (0.21–3.7)	0.855		
Smoking	3.86 (0.42–35.1)	0.231		
Chronic illnesses	6 (1.274–28.25)	0.023*	4.2 (0.82–21.5)	0.085
Usage of disease‐modifying agents	1.2 (0.21–7.11)	0.827		
Regular vaccination before splenectomy	1.33 (0.305–5.8)	0.702		
Regular vaccination after splenectomy	1.31 (0.21–5.5)	0.714		
Hb A1	0.99 (0.94–1.04)	0.567		
Hb A2	0.69 (0.39–1.2)	0.213		
Hb S	1.04 (0.99–1.1)	0.129		
Hb F	0.9 (0.86–1.03)	0.211		

Abbreviations: AOR, adjusted odds ratio; CI, confidence interval; Hb, hemoglobin.

* Significant at *p *≤ 0.05.

## 4 Discussion

Splenectomy is considered a universal therapeutic modality for several non‐malignant hematological diseases such as refractory immune thrombocytopenic, warm autoimmune hemolytic anemia, thalassemia, and SCD [12]. In SCD, the spleen is one of the first organs to be damaged, resulting in auto splenectomy and progressive functional asplenia rendering the patients at high risk of invasive bacterial infections. Interestingly, other patients with SCD do not fit the natural history of SCD and tend to have persistent splenomegaly. This translated into variability in the rate and the median age of splenectomy [13, 14]. In our study, the rate of splenectomy was significantly lower and performed at older ages as compared to the previous studies [11, 14]. Additionally, more than two‐thirds of splenectomies were performed electively with chronic left upper quadrant pain as the main indication, followed by asymptomatic large splenomegaly. Conversely, a prior acute sequestration crisis was the primary indication for splenectomy in other studies. Surprisingly, partial splenectomy was not utilized as an alternative to complete splenectomy, and only half of them were done laparoscopically, which is significantly lower than previously reported, probably due to a large spleen and severe adhesion [11, 15].

The spleen plays a crucial role in the prevention of morbidity and mortality among patients with SCD, as it is involved in the immune response and clearance of aberrant blood cells. On the other hand, splenectomy provides an immediate clinical benefit for those with recurrent acute splenic sequestration or severe hypersplenism translated into a reduction of hospitalizations and blood transfusions that could mitigate and reduce the alloimmunization risk [16, 17]. Our study findings are in keeping with previous ones and extended to the reduction of painful crises. Interestingly, individuals who underwent splenectomy before the age of 33 and those with comorbidity or chronic illness were more likely to have better outcomes and responses. Contrarily, risk thrombosis was more pronounced among participants who underwent splenectomy without clearly identifiable predictors or risk factors. However, thrombosis encompasses both arterial and venous systems, not confined to venous as previously reported [18, 19].

Acute chest syndrome (ACS) is the leading cause of mortality among patients with SCD, accounting for 25% of deaths. It is considered the second most common reason for hospitalization after vaso‐occlusive pain crisis in individuals with SCD. About 50% of patients with SCD will have at least one episode of ACS in their lifetime [20]. The pathogenesis of ACS is quite variable and includes infection, pulmonary vascular bed occlusion, and lung infarction. Previous reports indicate that the rate of death due to ACS is similar between splenectomized and non‐splenectomized individuals with SCD. Importantly, patients with SB^0^ had an increased risk of acute chest syndrome [21, 22]. Similarly, we found that the rate of ACS is significantly higher among splenectomized individuals however genotype is not identified. This could be attributed to poor immunization measures as more than one‐third of participants either did not receive vaccinations before splenectomy or were not on regular vaccinations thereafter.

This study emphasized that splenectomy plays a central role in the management of patients with SCD. Its impact extended beyond the reduction of blood transfusion and hospitalization but rather to reducing painful crises with maximum benefit among the young population and those with comorbidities though pathophysiology is unclear. However, the lack of randomized clinical trial and heterogenous clinical presentations create variability in clinical practice. In our study, the number of splenectomy procedures and the age at which they were done indicate that physicians prefer a conservative approach. Furthermore, participants were more likely to have persistent splenomegaly which could be attributed to the coexistence of beta‐thalassemia and increased prevalence of Arab Indian haplotypes in the eastern province of Saudi Arabia [23, 24]. Despite the fact that the majority of procedures were done electively, periprocedural vaccination practices are suboptimal. Additionally, beyond the augmented risk of post‐splenectomy acute chest syndrome, and venous thromboembolism, this study reveals that arterial events, particularly ischemic stroke occur at a similar rate too irrespective of being on hydroxyurea or having relatively high fetal hemoglobin. Unfortunately, familial history of thrombosis, the timing of events post‐splenectomy, and whether these events provoked or not are lacking and considered one of our study limitations. Another limitation is that our study is retrospective in nature and has a limited number of participants who underwent splenectomy though it is comparable. Moreover, our study did not address the rate of alloimmunization among those who underwent splenectomy in comparison to non‐splenectomised individuals, as this might alter splenectomy decision because of transfusion challenges. Lastly, participants' genotype status is crucial and should be incorporated into future research.

## 5 Conclusion

Splenectomy remains an effective therapeutic modality of treatment despite all advancements in the treatment paradigms for patients with SCD. However, short‐ and long‐term post‐splenectomy complications, heterogeneity of clinical presentations, and clinical response render decisions more complex. Therefore, incorporating patients' demographics, comorbidities, as well as genotypes might yield a more precise identification and selection of appropriate candidates for splenectomy. Additionally, physicians' orientation and patient's adherence to peri‐procedural immunizations are fundamental. Furthermore, identification of a high‐risk group of thrombosis may warrant post‐splenectomy antiplatelet or antithrombotic prophylaxis. Lastly, mitigating the risk of splenic complications while maintaining splenic function via partial splenectomy might be a future consideration.

## Author Contributions

**Mortadah Alsalman:** conceptualization, supervision, writing – original draft. **Zainab Bu‐Khamsin:** data curation and validation. **Fatimah AlSahaf:** data curation and validation. **Fatima Al Amer:** data curation and validation. **Fatema Alhelal:** data curation and validation. **Ali Essa:** methodology, formal analysis, writing – review and editing. **Mohammed Alessa:** conceptualization, writing – original draft, writing – review and editing. **Somaia Shehab‐Eldeen:** methodology, formal analysis, writing – original draft, writing – review and editing. **Abdallah Essa:** formal analysis, writing – review and editing.

## Funding

The authors have nothing to report.

## Conflicts of Interest

The authors declare no conflicts of interest.

## Transparency Statement

The lead author, Mortadah Alsalman, affirms that this manuscript is an honest, accurate, and transparent account of the study being reported; that no important aspects of the study have been omitted; and that any discrepancies from the study as planned (and, if relevant, registered) have been explained.

## Supporting information


Supporting File

## Data Availability

The data of this study are available from the first author upon reasonable request.
