# Supplementary material for: Splenectomy Impact and Outcome Among Patients With Sickle Cell Disease: A Cross‐Sectional Study
Source: Health Sci Rep. 2026 Jul 7;9(7):e72778. doi: 10.1002/hsr2.72778 (PMC13341955; doi:10.1002/hsr2.72778)
Supplement: Supplementary file 1 — Supporting File [file HSR2-9-e72778-s001.docx]

**Case Report Form (CRF)**

**Study: Splenectomy Impact and Outcome among Patients with Sickle Cell Disease**

**1. Participant Identification**

| Study ID Code | __________ |
| --- | --- |
| Hospital Record Number | __________ |
| Study Site | ☐ King Fahad Hospital ☐ Blood Disease Centre / KFU |

**2. Sociodemographic Data**

| Age (years) | _____ |
| --- | --- |
| Gender | ☐ Male ☐ Female |
| Marital status (optional) | ☐ Single ☐ Married ☐ Divorced ☐ Widowed |
| Chronic illness | ☐ Yes ☐ No If yes: ____________________ |

**3. Baseline SCD Characteristics (Pre-index / Baseline)**

| SCD genotype (Hb electrophoresis) | ☐ HbSS ☐ HbSC ☐ Other: ________ |
| --- | --- |
| Crises requiring hospitalization (per year) | _____ |
| ER visits (per year) | _____ |
| History of acute chest syndrome | ☐ Yes ☐ No |
| History of ICU admission | ☐ Yes ☐ No |
| History of stroke / venous thrombosis | Stroke: ☐ Yes ☐ No VTE: ☐ Yes ☐ No |

**4. Treatments & Transfusions**

| Blood transfusion history | ☐ Yes ☐ No |
| --- | --- |
| Transfusions per year | _____ |
| Hydroxyurea use | ☐ Yes ☐ No |
| If yes: duration & dose | Duration: ____ (months/years) Dose: ____ mg/day |

**5. Splenectomy (if applicable)**

| Splenectomy performed | ☐ Yes ☐ No |
| --- | --- |
| Age at splenectomy (years) | _____ |
| Type of surgery | ☐ Laparoscopic ☐ Open |
| Timing | ☐ Elective ☐ Emergency |
| Indication | ☐ Chronic LUQ pain ☐ Asymptomatic splenomegaly ☐ Multiple transfusions + LUQ pain ☐ Other: ________ |
| Vaccinations | Pre: ☐ Up to date ☐ Not up to date Post: ☐ Up to date ☐ Not up to date |

**6. Outcomes (Post-splenectomy / Follow-up)**

| Crises requiring hospitalization (per year) | _____ |
| --- | --- |
| Hospital admissions (per year) | _____ |
| ER visits (per year) | _____ |
| ICU admission | ☐ Yes ☐ No |
| Blood transfusions | ☐ Yes ☐ No Frequency/year: _____ |
| Complications | Stroke: ☐ Yes ☐ No VTE: ☐ Yes ☐ No Acute chest syndrome: ☐ Yes ☐ No |

**7. Laboratory Data (from EMR)**

| CBC | Hb: ____ g/dL WBC: ____ x10^9/L Platelets: ____ x10^9/L |
| --- | --- |
| LDH (U/L) | _____ |
| Creatinine (mg/dL) | _____ |
| Hb electrophoresis (%) | HbS: ____ HbF: ____ HbA2: ____ |
| Notes / abnormal results | ____________________________________________ |

**8. Clinical Response Classification (Splenectomy group)**

| ≥50% reduction in annual crises requiring hospitalization | ☐ Yes ☐ No |
| --- | --- |
| Response category | ☐ Good responder ☐ Poor responder |

**Case Report Form 1 completed by:**

**Name …………………………….……….. Signature ……………………..……………**

**Date ………………………………….…………..**
